# The priming effect of previous natural pandemic H1N1 infection on the immunogenicity to subsequent 2010-2011 influenza vaccination in children: a prospective cohort study

**DOI:** 10.1186/s12879-016-1769-7

**Published:** 2016-08-22

**Authors:** Eun Kyeong Kang, Byung Wook Eun, Nam Hee Kim, Jung Sub Lim, Jun Ah Lee, Dong Ho Kim

**Affiliations:** 1 Department of Pediatrics, Dongguk University Ilsan Hospital, Goyang, Korea; 2 Department of Pediatrics, Eulji University Eulji General Hospital, Seoul, Korea; 3 Department of Pediatrics, Inje University Ilsan Paik Hospital, Goyang, Korea; 4 Department of Pediatrics, Korea Cancer Center Hospital, Seoul, Korea

**Keywords:** Natural pandemic H1N1 infection, Influenza vaccines, Immunogenicity, Children

## Abstract

**Background:**

The effect of previous natural pandemic H1N1 (H1N1 pdm09) influenza infection on the immunogenicity to subsequent inactivated influenza vaccination in children has not been well studied. We aimed to evaluate the effect of H1N1 pdm09 natural infection and vaccination on the immunogenicity to subsequent 2010-2011 seasonal inactivated influenza vaccination in children.

**Methods:**

From October 2010 to May 2011, we conducted an open-label, multi-center study in children aged 6 months -18 years in Korea. We measured antibody titers with a hemagglutination-inhibition (HI) assay at baseline, 1 month, and 6 months after vaccination with trivalent split or subunit vaccines containing H1N1 pdm, A/H3N2, and B. The subjects were classified into 4 groups depending on the presence of laboratory-confirmed H1N1 pdm09 infection and/or vaccination in the 2009-2010 season; Group I: vaccination (-)/infection(-), Group II: vaccination (-)/infection(+), Group III: vaccination (+)/infection(-), Group IV: vaccination (+)/infection(+).

**Results:**

Among the subjects in group I, 47 subjects who had a baseline titer >1:10 were considered to have an asymptomatic infection. They were included into the final group II (*n* = 80). We defined the new group II as the infection-primed (IP) group and group III as the vaccine-primed (VP) group. Seroconversion rate (57.5 % vs 35.9 %, *p* = 0.001), seroprotection rate at 6 months after vaccination (70.8 % vs 61.8 %, *p* = 0.032), and GMT at 1 month after vaccination (129.9 vs 66.5, *p* = 0.002) were significantly higher in the IP group than in the VP group. In the 9–18 year-old group, seroconversion rate and immunogenicity at 1 and 6 months were significantly higher in the IP group than in the VP group. However in the 1–7 year-old age group, there was no significant difference between the two groups.

**Conclusions:**

Previous H1N1 pdm09 infection appears to have positive effects on immunogenicity of subsequent inactivated influenza vaccines against H1N1 pdm09 in older children.

## Background

After a worldwide pandemic influenza A H1N1(H1N1 pdm09) virus infection in 2009, seasonal influenza vaccines contain the A/California/7/2009 (H1N1)-like strain. Immunogenicity studies of pandemic H1N1 influenza vaccines and seasonal influenza vaccines including the A/California/7/2009 (H1N1)-like strain have been carried out globally. Although the pandemic influenza vaccines played some role in the elimination of H1N1 pdm09 influenza, H1N1 pdm09 influenza natural infection may contribute to the acquisition of herd immunity. The 2009 pandemic infection provided an opportunity to study the influence of natural infection on the immune responses. The evidence of a higher immune response elicited by natural H1N1 pdm09 infection than by vaccination was reported [1]. Furthermore, the longer duration of hemagglutination inhibition (HI) antibody by natural H1N1 pdm09 infection (mean lives of 11.8 months) than that by vaccination (mean lives of 8.35 months) was demonstrated [2]. On the contrary, most of the solid organ transplant recipients lack long-term humoral and cellular immune responses to influenza virus even after natural H1N1 pdm09 infection [3]. After primary influenza virus infection, the persistence of influenza virus-specific antibody-secreting cells and development of B-cell memory have been reported. This is probably the associated mechanism of long-lasting protection induced by natural infection [4].

Studies about the seroprevalence or immunogenicity in children are important because children are supposed to be the target and source of the spread of influenza infection [5, 6]. In children, one post-pandemic study compared the antibody titers against H1N1 pdm09 after natural infection and vaccination and demonstrated that vaccinated children exhibit lower HI titers compared to children with H1N1 pdm09 infection [7]. In our previous study, we similarly demonstrated that the immune response induced by natural H1N1 pdm09 infection was superior to that induced by pandemic H1N1 vaccination [8]. However, little is known about the effect of natural influenza infection on immunogenicity to subsequent inactivated influenza vaccination. We hypothesized that priming by previous natural influenza infection would have a more positive effect on the immunogenicity of vaccination in the following year than priming by previous vaccination. In this study, we aimed to evaluate the effect of H1N1 pdm09 natural infection and vaccination on the immunogenicity to subsequent 2010–2011 seasonal inactivated influenza vaccination containing the A/California/7/2009 (H1N1)-like strain in children.

## Methods

### Study subjects and questionnaire

From October 2010 to May 2011, we conducted an observational, open-label, prospective study in children at five hospitals located in Seoul and Gyeonggi Province in Korea. Healthy children aged 6 months to 18 years randomly received split or subunit vaccines according to vaccine assignment to each hospital (3 hospitals administered split vaccines and 2 hospitals administered subunit vaccines). Information about H1N1 pdm09 vaccination and laboratory-confirmed H1N1 pdm09 infection in the 2009–2010 season was obtained by a questionnaire. Confirmation of H1N1 pdm09 infection in that time period was obtained by H1N1 pdm09-specific real-time reverse transcriptase-polymerase chain reaction in most hospitals in Korea. An influenza A-positive result from the rapid antigen test in several subjects was also included because > 95 % of influenza isolates were H1N1 pdm09 viruses in Korea [9]. Subjects were divided into 4 groups depending on the presence of H1N1 pdm09 infection and/or H1N1 pdm09 vaccination in the 2009–2010 season; Group I: vaccination (-)/infection(-), Group II: vaccination (-)/infection(+), Group III: vaccination (+)/infection(-), Group IV: vaccination (+)/infection(+).

The demographic data were collected for analysis. Subjects who had severe allergy to influenza vaccine, acute febrile illness at the time of vaccination, received immunosuppressants including corticosteroids, history of transfusion within 6 months, and any other conditions which might interfere with the evaluation of the study were excluded. All subjects were observed for 30 min following vaccine administration to check for immediate local and/or systemic reactions. Parents/guardians were provided with diary cards to record the occurrence of solicited local reactions (pain, erythema, swelling), systemic symptoms (fever/chill, headache, myalgia, malaise) and unsolicited symptoms experienced during the first 7 days after vaccination. All adverse events (AEs) were reviewed during interviews with parents/guardians at the scheduled study visit on day 30 post-vaccination. Serious adverse events (SAEs) that occurred during the study were reported to the institutional review board (IRB). The study protocol was approved by the IRB of Korea Cancer Center Hospital (K-1001-001-013) and the IRB of each hospital, and written informed consent was obtained from parents of all subjects before participation in the study.

### Vaccines

Two kinds of vaccines were used in the study: Inflexal® V (subunit influenza vaccine, Crucell Company, Berna Biotech), SK influenza IX vaccine® (split influenza vaccine, Fluarix®, GlaxoSmithKline). Both vaccines contained the A/California/7/2009 (H1N1) strain (reassortant NYMC X-181), A/Victoria/210/2009(H3N2) strain (reassortant NYMC X-187), and B/Brisbane/60/2008 strain. The split vaccines were allocated to 2 hospitals and the subunit vaccines were allocated to the other 3 hospitals. Vaccines were injected into the deltoid muscle or the upper lateral thigh either as a single dose or as 2 doses with a 1-month interval for unprimed subjects younger than 9 years, as a dose of 0.25 ml for those aged 6 months to 3 years, or as a dose of 0.5 ml for those aged 3–18 years. We compared the immunogenicity of the two types of influenza vaccines among the children.

### Serological tests

Venous blood samples (5 ml) were collected at baseline, 1 month, and 6 months after vaccination with one or two doses in primed or unprimed subjects depending on the past influenza vaccination history. Anti-hemagglutinin antibody titers were determined using the HI test according to the WHO manual [10] using turkey erythrocytes for A/California/7/2009 (reassortant NYMC X-181, H1N1), and chicken erythrocytes for A/Victoria/210/2009 (reassortant NYMC X-187, H3N2) and B/Brisbane/60/2008, respectively. Immunogenicity was assessed according to the seroconversion rate, seroprotection rate, and geometric mean titer (GMT). Seroconversion was defined as a change versus baseline titer of <1:10 to a post-vaccination HI titer of ≥1:40 or a 4-fold or greater rise in titer in those with an initial HI titer of ≥1:10 and it was calculated at 1 month after vaccination. Seroprotection rate was defined as a HI titer of ≥1:40.

### Statistical analysis

Descriptive data are reported as numbers of subjects and as percentages or GMT, with 95 % confidence intervals (95 % CI). Comparisons of immunogenicity (seroprotection rate, seroconversion rate, GMT) among 4 groups were performed using the one-way ANOVA. Comparison between 2 groups was performed by Student’s *T*-test. *P* value of <0.05 was considered significant (2-tailed test). SPSS software version 13.0 (SPSS Inc., Chicago, IL, USA) was used for the statistical analyses.

## Results

### Study subjects

**Fig. 1:**
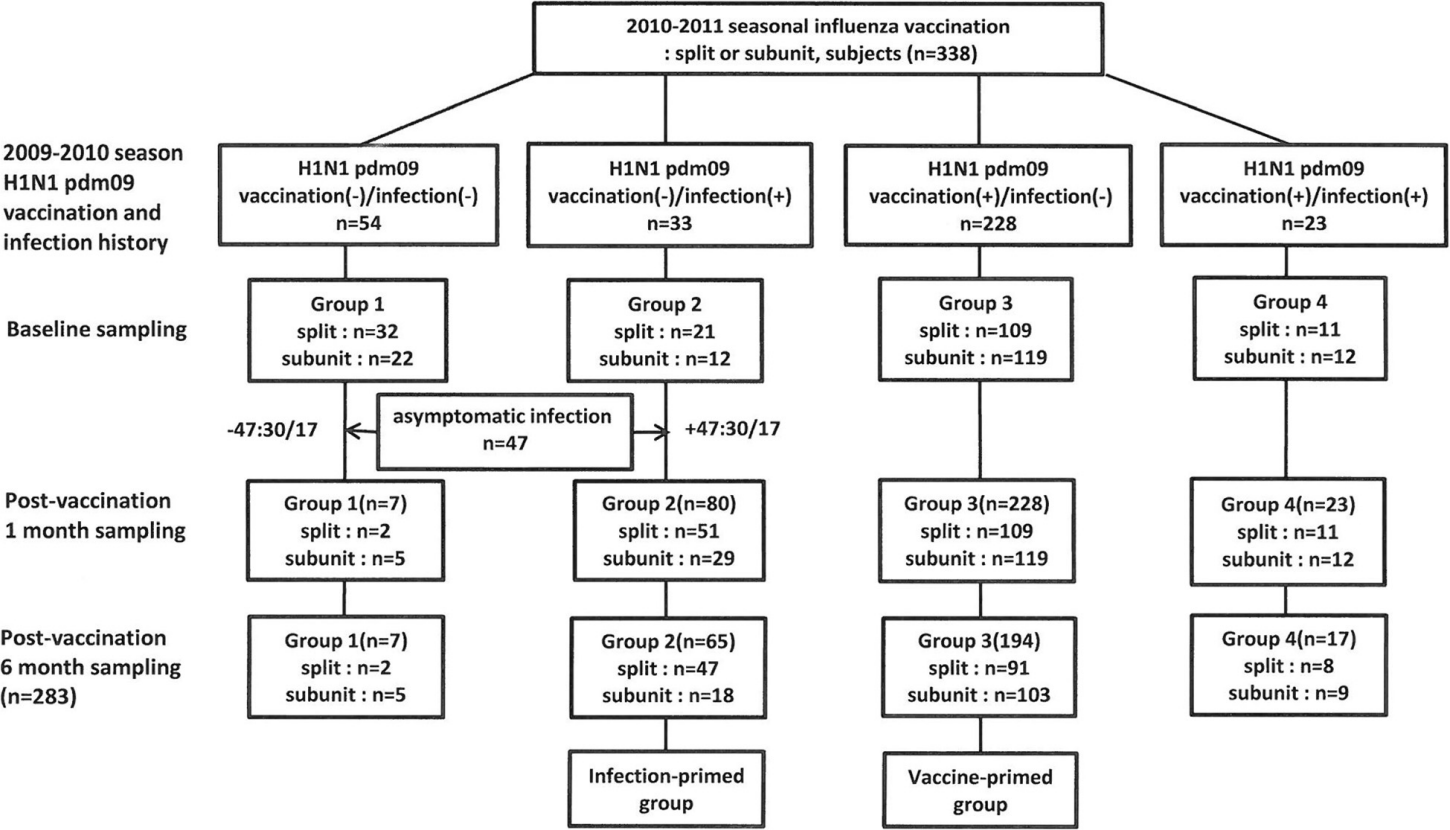
Overview of the clinical study

A total of 397 subjects were enrolled in this study, and paired samples were obtained from 338 subjects (baseline, 1 month after vaccination) for the evaluation of immunogenicity. The additional blood sample at 6 months after vaccination was taken from 283 subjects (Fig. 1). Among the subjects in group I, 47 subjects who had a baseline titer >1:10 were considered to have an asymptomatic infection. They were reclassified into the final group II (H1N1 pdm09 vaccination (-)/infection(+), *n* = 80). The number of subjects in the final group I was 7. We defined the new group II as the infection-primed (IP) group and group III as the vaccine-primed (VP) group (Fig. 1).

### Overall immunogenicity of influenza vaccines

**Fig. 2:**
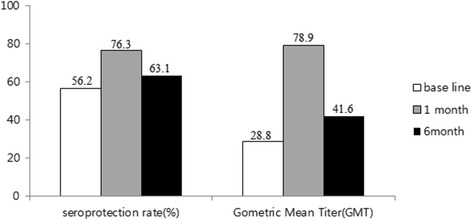
Seroprotection rate and geometric mean titer of antihaemagglutinin anibody to H1N1 pdm09 in total subjects at base line, 1 month, and 6 months after vaccination

The percentage of subjects with HI titer ≥1:40 against H1N1 pdm09 was 56.2, 76.3, and 63.1 % at baseline, 1 month, and 6 months after vaccination, respectively. The GMT of HI antibody against H1N1 pdm09 was 28.8, 78.9, and 41.6 at baseline, 1 month, and 6 months after vaccination, respectively. The GMT-fold increase for pH1N1 at 1 month after vaccination was 2.75 (Fig. 2).

### Comparison of immunogenicity of 2010–2011 influenza vaccines against H1N1 pdm09 between H1N1 pdm09 IP group and VP group

There was a significant difference in the seroprotection rate at 6 months after vaccination between the two groups (70.8 % vs 61.8 %, *p* = 0.032, Table 1). Seroconversion rate was significantly higher in the IP group than in the VP group (57.5 % vs 35.9 %, *p* = 0.001). GMT at 1 month after vaccination was higher in the IP group than in the VP group (129.9 vs 66.5, *p* = 0.002). GMT at 6 months after vaccination was higher in the IP group than in the VP group with no statistical significance (54.5 vs 37.1, *p* = 0.150). However, mean age (10.0 yr vs 8.1 yr, *p* = 0.001) and split/subunit ratio (*p* = 0.013) were significantly different between the two groups. When we compared the two groups after dividing them into two age groups (1–7 year-old group and 9–18 year-old group), after excluding the 8-year-old subjects, there were no differences in mean age and proportion of two vaccine types (split vs subunit) between the IP and VP groups. In the 9–18 year-old group (Table 2), seroconversion rate and immunogenicity at 1 and 6 months were significantly different between the IP and VP groups. However in the 1–7 year-old age group, there was no significant difference between the two groups (Table 3).

**Table 1:** Comparison of immunogenicity of 2010-2011 influenza vaccines against H1N1 pdm09 between infection-primed group and vaccine-primed group among the total subjects

		IP (*n* = 80)	VP (*n* = 228)	*P* value
Split/subunit ratio		51/29	109/119	0.013
Mean age		8.06 (±4.28)	10.02 (±3.88)	0.001
Seroprotection (%)	Baseline	58.75 (47/80)	54.82 (125/228)	0.544
	1 month	80.0 (64/80)	75.00 (171/228)	0.367
	6 months	70.77 (46/65)	61.86 (120/194)	0.032
Seroconversion (%)		57.5 (46/80)	35.96 (82/228)	0.001
GMT (95 % CI)	Baseline	33.93 (29.80–38.05)	27.43 (24.69–30.18)	0.109
	1 month	129.96 (99.18–160.74)	66.46 (58.15–72.76)	0.002
	6 months	54.50 (42.32–66.67)	37.11 (33.30–40.92)	0.150

**Table 2:** Comparison of immunogenicity of 2010-2011 influenza vaccines against H1N1 pdm09 between infection-primed group and vaccine-primed group in children 9-18 years of age

		IP (*n* = 32)	VP (*n* = 145)	*P* value
Split/subunit ratio		17/15	57/88	0.152
Mean age		12.57 (±2.52)	12.22 (±2.22)	0.432
Seroprotection (%)	Baseline	71.88 (23/32)	57.24 (83/145)	0.098
	1 month	93.75 (30/32)	73.79 (107/145)	0.001
	6 months	78.26 (18/23)	57.03 (73/128)	0.038
Seroconversion (%)		68.75 (22/32)	37.24 (54/145)	0.001
GMT (95 % CI)	Baseline	45.55 (36.80–54.29)	29.18 (25.51–32.84)	0.041
	1 month	246.75 (177.61–315.89)	69.98 (59.69–80.26)	0.000
	6 months	101.81 (65.66–137.96)	39.57 (34.91–44.22)	0.001

**Table 3:** Comparison of immunogenicity of 2010-2011 influenza vaccines against H1N1 pdm09 between infection-primed group and vaccine-primed group in subjects less than 8 years of age

		IP (*n* = 46)	VP (*n* = 66)	*P* value
Split/subunit ratio		32/14	44/22	0.749
Mean age		4.90 (±1.76)	5.17 (±1.77)	0.414
Seroprotection (%)	Baseline	47.83 (22/46)	50.0 (33/66)	0.823
	1 month	69.57 (32/46)	78.79 (52/66)	0.283
	6 months	65.85 (27/41)	56.00 (28/50)	0.344
Seroconversion (%)		47.83 (22/46)	40.91 (27/66)	0.472
GMT (95 % CI)	Baseline	27.03 (22.77–31.29)	24.67 (20.36–28.99)	0.620
	1 month	80.00 (62.62–97.38)	61.53 (50.70–72.35)	0.392
	6 months	37.38 (30.03–44.74)	31.17 (24.22–38.11)	0.481

### Comparison of immunogenicity of split and subunit influenza vaccines against H1N1 pdm09

In the split vaccine group which included subjects who were 9–18 year-old, seroconversion rate, seroprotection rate, and GMT at 1 and 6 months were significantly higher in the IP group than in the VP group (Table 4). In the subunit vaccine group which included subjects who were 9–18 year-old, seroconversion rate and seroprotection rate at 1 month were significantly higher in the IP group than in the VP group (Table 4). Immunogenicity to split vaccines at 6 months after vaccination was significantly better than that to subunit vaccines in the VP group (% of subjects with HI antibody ≥1:40, 76.00 vs 45.68 and GMT 64.09 vs 29.05, *P* < 0.01).

**Table 4:** Comparison of immunogenicity of 2010-2011 influenza vaccines against H1N1 pdm09 between infection-primed group and vaccine-primed group in the split and subunit vaccine group among children 9-18 years of age

		IP (*n* = 32)	VP (*n* = 145)	*P* value
Split		17	57	
Mean age		12.81 (±2.44)	11.76 (±2.01)	0.077
Seroprotection (%)	Baseline	70.59 (12/17)	52.63 (30/57)	0.098
	1 month	94.12 (16/17)	75.44 (43/57)	0.001
	6 months	86.67 (13/15)	76.00 (38/50)	0.038
Seroconversion (%)		64.71 (11/17)	38.60 (22/57)	0.001
GMT (95 % CI)	Baseline	35.39 (26.93–43.86)	30.61 (23.99–37.24)	0.644
	1 month	250.56 (138.16–362.95)	77.13 (66.37–87.90)	0.004
	6 months	139.29 (94.10–184.47)	64.09 (53.03–75.14)	0.019
Subunit		15	88	
Mean age		12.30 (±2.67)	12.74 (±2.57)	0.544
Seroprotection (%)	Baseline	73.33 (11/15)	60.23 (53/88)	0.345
	1 month	93.33 (14/15)	73.86 (65/88)	0.022
	6 months	62.5 (5/8)	45.68 (37/81)	0.369
Seroconversion (%)		73.33 (11/15)	37.50 (33/88)	0.011
GMT (95 % CI)	Baseline	60.63 (36.22–85.04)	28.28 (23.98–32.59)	0.015
	1 month	242.51 (158.95–326.08)	65.70 (51.58–79.82)	0.002
	6 months	56.57 (10.48–102.66)	29.05 (23.28–34.82)	0.166

### Vaccine safety

Both vaccines were safe and no SAEs occurred. Total solicited and unsolicited AEs were not reported differently between the split and subunit vaccine groups (65 vs 58, 11 vs 9, *p* > 0.05). Local AEs occurred more frequently after administration of split vaccine (53 vs 25, *p* < 0.05) (Table 5).

**Table 5:** Incidence of adverse events (AEs) during the first 7 days after vaccination

		Split vaccine (*n* = 181)	Subunit vaccine (*n* = 190)	*p*-value
Solicited AE	(Event/subject)	65/42	58/34	0.26/0.21
Systemic AE	(Event/subject)	12/10	33/24	0.066/0.018
Fever/chill		3	9	0.094
Headache		1	7	0.038
Myalgia		6	13	0.12
Malaise		2	4	0.45
Local AE	(Event/subject)	53/36	25/16	0.006/0.001
Pain		31	14	0.004
Erythema		5	2	0.23
Swelling		9	9	0.92
Unsolicited AE		11	9	0.57

## Discussion

The present study demonstrated that previous H1N1 pdm09 natural influenza infection has superior priming effects compared to previous H1N1 pdm09 vaccination on immunogenicity to subsequent inactivated influenza vaccination containing the A/California/7/2009 (H1N1)-like strain in the 9–18 year-old group. The results of our study are consistent with those in the study that showed that priming by natural infection was important to elicit a strong immune response after inactivated vaccination in young children [11]. An animal study showed that pigs infected with influenza virus mount an effective immune response and are protected from subsequent challenge, whereas the inactivated-virus vaccine does not consistently confer complete protection to challenge [12]. One study performed in Hong Kong showed that children who had prior seasonal influenza infection had significantly lower risk of subsequent pandemic influenza infection, whereas in children who received prior seasonal influenza vaccination, this protective effect was reduced [13]. Cross-protection via mechanisms associated with cell-mediated immunity has been suggested. These studies showed that influenza virus natural infection induced better protective immunity than inactivated influenza vaccine.

In terms of immune responses, live-attenuated influenza vaccines (LAIV) mimic natural infection. LAIV can induce mucosal immune and cellular immune responses [14]. From the results of the present study, we may suppose that inactivated influenza vaccination following live influenza vaccination will induce better immunogenicity than vaccination with inactivated vaccines for two successive years. Furthermore, the possibility of downside of yearly vaccination with inactivated vaccines against seasonal influenza viruses in young children was suggested in the aspect of preventing the induction of heterosubtypic immunity [15]. One small clinical study performed before pandemic influenza infection in 2009 demonstrated that prime/boost combinations of LAIV and trivalent inactivated influenza vaccine in young children induced similar humoral responses, and regimens containing LAIV induced T-cells are relevant for heterosubtypic immunity [16]. T cells can target internal proteins common to heterologous influenza viral strains, and cytotoxic T lymphocytes help to reduce the severity of disease or complications [17]. Especially in children, adequate stimulation of cellular immunity may be important for reducing severe outcomes in case of future pandemics.

In the present study, immunogenicity to split vaccines was significantly better than that to subunit vaccines at 6 months after vaccination in the VP group. In addition, the priming effect induced by infection was markedly greater in the split vaccines group than in the subunit vaccines group in 9–18 year-old children. In our previous study, the immunogenicity of split vaccines appeared to be better than that of subunit vaccines in unprimed children younger than 3 years in Korea [18]. This might be due to the difference in influenza antigens present in the two types of vaccines, and concretely, internal virus proteins (nucleoprotein and matrix protein) were detected in some split vaccines [19, 20].

In a study assessing the efficacy of inactivated vaccines against influenza A infection, efficacy was higher in the 6–15 years age group than in the 1–5 years age group [21]. In this study, previous influenza infection was associated with increased antibody responses to inactivated vaccination against H1N1 pdm09 in children 9–18 years of age than in children less than 8 years of age. Priming effect induced by previous influenza infection seemed to be prominent in the older children. In contrast, previous vaccination was associated with reduced antibody responses to inactivated influenza vaccines against seasonal influenza A in children 9–17 years of age [22]. Both age and history of previous vaccination or infection seemed to influence the immunogenicity of influenza vaccination.

In this study, asymptomatic or subclinical infection was suspected in many subjects who were not vaccinated or infected. We initially classified only laboratory-confirmed cases to the infection group via questionnaires, excluding the physician’s tentative clinical diagnosis. Thus, we might have underestimated the proportion of infected subjects. Therefore, among the subjects in group I, we reclassified 47 subjects who had a baseline HI titer > 1:10 and who were not vaccinated or infected into group II. They were considered to have an infection because they did not have preexisting cross-reactive antibodies [23]. Among subjects in the vaccination (+)/infection (-) group, there might be a possibility of an asymptomatic infection.

There are several limitations to this study. First, there is some possibility of inaccurate information obtained from the questionnaire about previous H1N1 pdm09 infection and vaccination. However, memory error is less likely because novel pandemic H1N1 influenza in Korea was a hot issue at that time especially among caregivers. Another limitation is that we did not investigate the history of taking oseltamivir among the children who had natural infection. Oseltamivir treatment may reduce the viral load of patients with infection [24] and may negatively influence the immune response against the influenza virus.

## Conclusions

In conclusion, previous H1N1 pdm09 natural infection had a superior priming effect on immunogenicity to subsequent inactivated influenza vaccination in the 9–18 year-old age group. We can suppose that initial live attenuated vaccines may induce a more effective immune response to subsequent inactivated vaccines in unprimed children.

## Abbreviations

CI: confidence interval
GMT: geometric mean titer
HI: hemagglutination-inhibition
IP: infection-primed
LAIV: live-attenuated influenza vaccines
SAEs: serious adverse events
VP: vaccine-primed

## References

[1] Chan KH, To KK, Hung IF, Zhang AJ, Chan JF, Cheng VC (2011). Differences in antibody responses of individuals with natural infection and those vaccinated against pandemic H1N1 2009 influenza. Clin Vaccine Iimmunol.

[2] Liu W, Ma MJ, Tang F, He C, Zhang XA, Jiang LF (2012). Host immune response to A(H1N1)pdm09 vaccination and infection: a one-year prospective study on six cohorts of subjects. Vaccine.

[3] Baluch A, Humar A, Egli A, Gubbay J, Lisboa L, Wilson L et al. Long term immune responses to pandemic influenza A/H1N1 infection in solid organ transplant recipients. PLoS One. 2011; doi:10.1371/journal.pone.0028627. 10.1371/journal.pone.0028627 PMC3237471 22194870

[4] Jones PD, Ada GL (1987). Persistence of influenza virus-specific antibody-secreting cells and B-cell memory after primary murine influenza virus infection. Cell Immunol.

[5] Reichert TA, Sugaya N, Fedson DS, Glezen WP, Simonsen L, Tashiro M (2001). The Japanese experience with vaccinating schoolchildren against influenza. N Engl J Med.

[6] Miller E, Hoschler K, Hardelid P, Stanford E, Andrews N, Zambon M (2010). Incidence of 2009 pandemic influenza A H1N1 infection in England: a cross-sectional serological study. Lancet.

[7] von Kries R, Weiss S, Falkenhorst G, Wirth S, Kaiser P, Huppertz HI et al. Post-pandemic seroprevalence of pandemic influenza A (H1N1) 2009 infection (swine flu) among children <18 years in Germany. PLoS One 2011; doi:10.1371/journal.pone.0023955. 10.1371/journal.pone.0023955 PMC3168498 21915270

[8] Kang EK, Lim JS, Lee JA, Kim DH (2013). Comparison of immune response by virus infection and vaccination to 2009 pandemic influenza A/H1N1 in children. J Korean Med Sci.

[9] Korea Centers for Disease Control and Prevention (2009). Current status of selected infectious diseases. Public Health Wkly Rep.

[10] World Health Organization. WHO manual on animal influenza diagnosis and surveillance. http://www.who.int/csr/resources/publications/influenza/en/whocdscsrncs20025rev.pdf. Accessed 27 Nov 2011.

[11] El-Madhun AS, Cox RJ, Soreide A, Olofsson J, Haaheim LR (1998). Systemic and mucosal immune responses in young children and adults after parenteral influenza vaccination. J Infect Dis.

[12] Larsen DL, Karasin A, Zuckermann F, Olsen CW (2000). Systemic and mucosal immune responses to H1N1 influenza virus infection in pigs. Vet Microbiol.

[13] Cowling BJ, Ng S, Ma ES, Cheng CK, Wai W, Fang VJ (2010). Protective efficacy of seasonal influenza vaccination against seasonal and pandemic influenza virus infection during 2009 in Hong Kong. Clin Infect Dis.

[14] Kreijtz JH, Fouchier RA, Rimmelzwaan GF (2011). Immune responses to influenza virus infection. Virus Res.

[15] Bodewes R, Kreijtz JH, Rimmelzwaan GF (2009). Yearly influenza vaccinations: a double-edged sword?. Lancet Infect Dis.

[16] Hoft DF, Babusis E, Worku S, Spencer CT, Lottenbach K, Truscott SM (2011). Live and inactivated influenza vaccines induce similar humoral responses, but only live vaccines induce diverse T-cell responses in young children. J Infect Dis.

[17] Thomas PG, Keating R, Hulse-Post DJ, Doherty PC (2006). Cell-mediated protection in influenza infection. Emerg Infect Dis.

[18] Kim YK, Eun BW, Kim NH, Kang EK, Lee BS, Kim DH (2013). Comparison of immunogenicity and reactogenicity of split versus subunit influenza vaccine in Korean children 6 through 35 months of age. Scand J Infect Dis.

[19] Chaloupka I, Schuler A, Marschall M, Meier-Ewert H (1996). Comparative analysis of six European influenza vaccines. Eur J Clin Microbiol Infect Dis.

[20] Cox RJ, Brokstad KA (1999). The postvaccination antibody response to influenza virus proteins. APMIS.

[21] Neuzil KM, Dupont WD, Wright PF, Edwards KM (2001). Efficacy of inactivated and cold-adapted vaccines against influenza A infection, 1985 to 1990: the pediatric experience. Pediatr Infect Dis J.

[22] Ng S, Ip DK, Fang VJ, Chan KH, Chiu SS, Leung GM, Peiris JS, Cowling BJ. The effect of age and recent influenza vaccination history on the immunogenicity and efficacy of 2009-10 seasonal trivalent inactivated influenza vaccination in children. PLoS One. 2013; doi:10.1371/journal.pone.0059077. 10.1371/journal.pone.0059077 PMC3595209 23554974

[23] Hancock K, Veguilla V, Lu X, Zhong W, Butler EN, Sun H (2009). Cross-reactive antibody responses to the 2009 pandemic H1N1 influenza virus. N Engl J Med.

[24] Li IW, Hung IF, To KK, Chan KH, Wong SS, Chan JF (2010). The natural viral load profile of patients with pandemic 2009 influenza A(H1N1) and the effect of oseltamivir treatment. Chest.

